# N-glycosylation of the envelope glycoprotein I is essential for the proliferation and virulence of the duck plague virus

**DOI:** 10.1186/s13567-024-01398-4

**Published:** 2024-10-26

**Authors:** Yaru Ning, Mingshu Wang, Anchun Cheng, Qiao Yang, Bin Tian, Xumin Ou, Di Sun, Yu He, Zhen Wu, Xinxin Zhao, Shaqiu Zhang, Ying Wu, Juan Huang, Yanling Yu, Ling Zhang, Renyong Jia, Mafeng Liu, Dekang Zhu, Shun Chen

**Affiliations:** 1 Institute of Veterinary Medicine and Immunology, Sichuan Agricultural University, Chengdu, 611130 China; 2 Research Center of Avian Disease, College of Veterinary Medicine, Sichuan Agricultural University, Chengdu, 611130 China; 3 Key Laboratory of Animal Disease and Human Health of Sichuan Province, Chengdu, 611130 China; 4 International Joint Research Center for Animal Disease Prevention and Control of Sichuan Province, Chengdu, 611130 China; 5 Engineering Research Center of Southwest Animal Disease Prevention and Control Technology, Ministry of Education of the People’s Republic of China, Chengdu, 611130 China

**Keywords:** Duck plague virus, virulence gene, glycoprotein I, N-glycosylation, pathogenicity

## Abstract

Duck plague virus (DPV) causes the highly pathogenic duck plague, and the envelope glycoprotein I (gI), as one of the key virulence genes, has not yet had its critical virulence sites identified through screening. This study used reverse genetics technology to target the gI, specifically within the DPV genome. Four DPV mutants with gI N-glycosylation site mutations were designed and constructed, and these mutant strains were successfully rescued. Our results confirmed that three asparagine residues of gI (N_69_, N_78_, and N_265_) are N-glycosylation sites, and western blot analysis substantiated that glycosylation at each predicted N-glycosylation site was compromised. The deglycosylation of gI leads to the protein misfolding and subsequent retention in the endoplasmic reticulum (ER). The subsequent deglycosylated gI is carried into the Golgi apparatus (GM130) in the interaction of gE. Compared to the parental virus, the mutated virus shows a 66.3% reduction in intercellular transmission capability. In ducks, the deglycosylation of gI significantly reduces DPV replication in vivo, thereby weakening the virulence of DPV. This study represents the first successful creation of a weak DPV virus strain by specific mutation at the N-glycosylation site. The findings provide a foundational understanding of DPV pathogenesis and form the basis for developing live attenuated vaccines against the disease.

## Introduction

Duck plague (DP), also known as duck viral enteritis (DVE) [1], is an acute and highly contagious septicaemic disease. It is characterised by vascular damage, tissue haemorrhage, erosion of the gastrointestinal mucosa, impairment of lymphoid organs, and degenerative changes in solid organs. This disease affects ducks, geese, and other Anseriformes and is caused by duck plague virus (DPV) [2, 3]. China produces over 4 billion ducks annually, accounting for approximately 70% of the global output. DP is one of the most destructive diseases for the global waterfowl breeding industry, affecting many species. The incidence and mortality rates of DP can reach up to 95%, leading to significant economic losses and severely impacting the healthy development of the waterfowl breeding sector [2, 4]. Immunosuppression and latent infections resulting from DPV infection complicate prevention and control strategies that are used for the disease [5–7].

DPV belongs to the order *Herpesvirales*, family *Herpesviridae*, subfamily *Alphaherpesvirinae*, and genus Marek’s disease virus. Mature DPV virions are spherical and relatively large, with a diameter ranging from 150 to 300 nm. The structure of DPV is complex, consisting of a core formed by the intertwining of double-stranded DNA and nucleoproteins, an icosahedral capsid structure made of proteins, a tegument layer also composed of proteins, and an outer envelope containing glycoproteins. The DPV CHv strain is a highly virulent virus with a genomic full length of 162 175 base pairs (bp), which includes 78 potential open reading frames (ORFs) encoding functional proteins [8]. The genomic structure consists primarily of a long unique region (UL), a short unique region (US), and internal repeats (IR) that flank the US region. The genome is arranged as 5′-UL-IRS-US-TRS-3′. In DPV research, multiple genes encoding envelope proteins have been identified. These envelope proteins are essential for the virus life cycle and pathogenicity. Specifically, the UL1, UL10, UL22, UL27, UL44, UL49.5, UL53, and US4-8 genes encode envelope proteins [9–12]. The envelope glycoprotein I (gI), encoded by the US7 gene, has been recognised as a crucial virulence factor for DPV. This finding was based on clinical symptom observations and gross pathological analysis of ducks infected with DPV△gI. Because of gI’s significant role in viral pathogenicity, it has become a potential target for studying the mechanisms of viral attenuation [13, 14]. However, the molecular mechanisms by which gI is involved in DPV attenuation have not yet been reported. This indicates that there is still a knowledge gap in the field of DPV research.

Viral pathogens require the proper expression and localisation of virulence genes to ensure successful infection. Like many proteins, virulence genes must undergo post-translational modifications to be correctly localised, secreted, and functional. N-glycosylation is a common post-translational modification that assists in the proper folding of newly synthesised proteins [15–17]. Proteins recognise polysaccharides through a specific consensus sequence N-X-T/S. Glycosyltransferases catalyse the attachment of polysaccharides to asparagine residues at specific sequences [18, 19]. Abnormal glycosylation can lead to reduced virulence in both animal and plant pathogens [20]. The Rotavirus (RV) non-structural protein 4 (NSP4) undergoes deglycosylation, which causes NSP4 to be mislocalised. This process leads to a delayed increase in cytosolic calcium ion concentration, a reduction in virus formation, and a significant decrease in the replication capacity of the glycosylation-deficient virus in various cell lines. This reduction ranges from 10 to 10 000-fold compared to the wild-type virus. Additionally, the pathogenicity of the glycosylation-deficient virus in mice is lower than that of the wild-type virus [21]. N-glycosylation modifications highly characterise the hemagglutinin (HA) protein of the influenza virus [22]. Removal of N-glycans from epitopes inhibits the recognition of HA by specific antibodies, thus helping the virus evade the host's humoral immune response [23, 24]. Typically, viruses increase their virulence by manipulating or evading the host's immune response [25, 26]. Herpesvirus glycoproteins gB, gH, gD, gJ, and gM are all connected via asparagine-linked glycosylation, which plays an essential role in their proper folding, transport, and immune evasion [9, 27–31]. However, the precise role of DPV gI in viral proliferation and virulence within its natural host requires further investigation.

In this study, we comprehensively evaluated the functionality of all potential N-glycosylation sites on the DPV gI. We showed that N-glycosylated gI plays a critical role in organelle transport. When gI is deglycosylated, it loses its ability to move within host cells. Additionally, deglycosylation of gI significantly reduced the intercellular spread of the mutant virus. In ducks, deglycosylation of gI notably decreases the replication of DPV in vivo.

## Materials and methods

### Cells and viruses

Duck embryonic fibroblasts (DEFs) were derived from nine-day-old duck embryos and stored in Eagle’s Minimum Essential Medium (MEM) (Gibco, Shanghai, China) supplemented with 10% Newborn Bovine Serum (NBS) (Gibco, MD, USA). HEK293T cells are maintained in our laboratory and preserved in 1640 Medium (Gibco, Shanghai, China) containing 10% Foetal Bovine Serum (FBS) (Gibco, MD, USA). All cells were cultured at 37 °C with 5% CO_2_. The Chinese strong strain DPV CHv (GenBank login number: JQ647509) has been sequenced for 50 generations of cytotoxic DPV CHv_50_ in DEFs. This strain was preserved and provided by the Institute of Immunology, College of Veterinary Medicine, Sichuan Agricultural University.

### Plasmids

Recombinant plasmids were designed based on the eukaryotic vector pCAGGS. These plasmids include pCAGGS-gI-Flag, pCAGGS-gI_N69A_-Flag, pCAGGS-gI_N78A_-Flag, pCAGGS-gI_N265A_-Flag, and pCAGGS-gI_N69/78/265A_-Flag, for the expression of recombinant gI. In summary, the gI gene fragment was amplified from the DPV CHv strain, and a Flag tag was introduced at its C-terminus and cloned into the *EcoR* I and *Bgl* II restriction enzyme-linearised pCAGGS vector. Using the oligonucleotide primers shown in Table 1, different N-glycosylation site mutations were introduced into the gI gene by standard overlapping PCR.

**Table 1:** **Primer sequences for overlapping PCR and RT-qPCR**

Name	Purpose	Sequence (5′–3′)
pCAGGS-gI-Flag-F	Construct pCAGGS-gI-Flag plasmid	5ʹ- CATCATTTTGGCAAAGAATTCGCCACCATGGGAACGACACGACATATACTGATA-3ʹ
pCAGGS-gI-Flag-R		5ʹ-TTGGCAGAGGGAAAAAGATCTTTACTTGTCATCGTCGTCCTTGTAGTCCTTGTCATCGTCGTCCTTGTAGTCTTCTGTTTTATGATCCCCAGA-3ʹ
pCAGGS-gI_N69A_-Flag-F	overlapping PCR	5ʹ-CTACCGCCAGTATACGCGGGAACTGTCGAGCTA-3ʹ
pCAGGS-gI_N69A_-Flag-R		5ʹ-CGCGTATACTGGCGGTAGTTCGGCCTGTGATCC-3ʹ
pCAGGS-gI_N78A_-Flag-F	overlapping PCR	5ʹ-GAGCTACTAGTATATGCGATTTCTCGTCACTGC-3ʹ
pCAGGS-gI_N78A_-Flag-R		5ʹ-CGCATATACTAGTAGCTCGACAGTTCCATTGTA-3ʹ/ 5ʹ-CGCATATACTAGTAGCTCGACAGTTCCCGCGTA-3ʹ
pCAGGS-gI_N265A_-Flag-F	overlapping PCR	5ʹ-ATGCAGCCTACGCATGCGGATACCAATAGTGGA-3ʹ
pCAGGS-gI_N265A_-Flag-R		5ʹ-CGCATGCGTAGGCTGCATATCATCAGATAATGT-3ʹ
pCAGGS-gI_N69/78/265A_-Kan-F	The Kan fragment was introduced into the recombinant plasmid	5ʹ-GACGACGATGACAAGTAAAGATCTTTCACATCAGTATGACTAAAAGTAAATGTTTTTGTTATGATTGACTGTTTTAGGGATAACAGGGTAATCGATTT-3ʹ
pCAGGS-gI_N69/78/265A_-Kan-R		5‘-TTGGCAGAGGGAAAAAGATCTGCCAGTGTTACAACCAAT-3ʹ
gI-repair-F	Amplified complement fragments	5ʹ-CGTACTTCCAGTATTGTCCAGTGCGCCATATAGACGATATATTGAGTTTCAAAAATAGAAATGGGAACGACACGACATATA-3ʹ
gI-Kan- repair-R		5ʹ-TACAAATATATTTGGATGTTAATGAAAGGCAAACAGTCAATCATAACAAAAACATTTACTTTTAGTCATACTGATGTGAAGCCAGTGTTACAACCAAT-3ʹ
UL30-F	RT-qPCR	5ʹ-TTTTCCTCCTCCTCGCTGAGT-3ʹ
UL30-R		5ʹ-GGCCGGGTTTGCAGAAGT-3ʹ

Moreover, we have introduced the “Kan” fragment into the aforementioned five recombinant vectors to create infectious clones containing gI mutations. Using pCAGGS-gI-Flag as a model, we used the oligonucleotide primers listed in Table 1 to amplify the “Kan” fragment from the pEP-Kan-S vector. We then cloned this fragment into the *Bgl* II restriction enzyme-linearised pCAGGS-gI-Flag vector, creating the pCAGGS-gI-Flag-Kan recombinant plasmid.

### Construction and rescue of recombinant viruses

The infectious clones pDPV CHv△gI and pDPV CHv△gI + △gE, which are deletions of the DPV CHv strain, were provided by our laboratory. These clones were used to create the following gI mutant infectious clones: pDPV CHv-gI_N69A_, pDPV CHv-gI_N78A_, pDPV CHv-gI_N265A_, pDPV CHv-gI_N69/78/265A_, pDPV CHv-gI_N69/78/265A_ repair, and pDPV CHv△gE-gI_N69/78/265A_. The steps are based on creating gene restoration strains of gene-deletion viruses using the oligonucleotide primers listed in Table 1.

To rescue the modified virus, plasmids were extracted from the gI mutant infectious clones and then transfected into DEFs cultured in 12-well plates. The transfected cells showed visible fluorescence (EGFP) and cytopathic effect (CPE), and the cell supernatant was collected and stored as viral stock solution. The recombinant viruses were all identified through sequencing. The correctly identified recombinant viruses were designated as DPV CHv-gI_N69A_, DPV CHv-gI_N78A_, DPV CHv-gI_N265A_, DPV CHv-gI_N69/78/265A_, DPV CHv-gI_N69/78/265A_ repair, and DPV CHv∆gE-gI_N69/78/265A_.

### Viral plaque assay

DEFs on six-well plates were infected with DPV CHv_50_ or recombinant viruses at a multiplicity of infection (MOI) of 0.0001. After a 2-h adsorption period, the virus solution used to inoculate the cells was removed, and any remaining virus on the cell surface was washed away. The DEFs were then overlaid with MEM containing 1% FBS and 2% methylcellulose (Solarbio, Beijing, China). Five days post-infection, the MEM with methylcellulose was removed, and the cells were fixed with 4% paraformaldehyde (Solarbio, Beijing, China) for 20 min. The cells were then stained with 0.5% crystal violet (Solarbio, Beijing, China) for 5 min. After several washes with PBS, the visible plaques were photographed.

### Western blot analysis of recombinant gI

Plasmids pCAGGS-gI-Flag, pCAGGS-gI_N69A_-Flag, pCAGGS-gI_N78A_-Flag, pCAGGS-gI_N265A_-Flag, and pCAGGS-gI_N69/78/265A_-Flag were transfected into DEFs growing in 12-well plates at a quantity of 1000 ng per well. After 36 h, the cells were lysed using RIPA buffer (Beyotime, China, P0013D). The lysates were centrifuged at 4 °C and 12 000 rpm for ten minutes to obtain a clarified supernatant. The supernatant was mixed with the sample buffer and boiled at 100 °C for 10 min. This mixture was then subjected to 12% sodium dodecyl sulphate–polyacrylamide gel electrophoresis (SDS-PAGE), followed by western blot. For western blot detection, the primary antibodies used were anti-Flag monoclonal antibody (mAb) (M185-3L, MBL) and GAPDH (60 004–1-lg, Proteintech). The secondary antibody was horseradish peroxidase (HRP)-conjugated goat anti-mouse IgG (SA00001-1, Proteintech). Visualisation was carried out using the Clarity Western ECL system (Bio-Rad, USA).

### Deglycosylation of recombinant gI

1000 ng of plasmid DNA per well, including pCAGGS-gI-Flag, pCAGGS-gI_N69A_-Flag, pCAGGS-gI_N78A_-Flag, pCAGGS-gI_N265A_-Flag and pCAGGS-gI_N69/78/265A_-Flag, was transfected into DEFs cultured in a 12-well plate. At a MOI of 0.01, the recombinant viruses DPV CHv_50_, DPV CHv-gI_N69A_, DPV CHv-gI_N78A_, DPV CHv-gI_N265A_, and DPV CHv-gI_N69/78/265A_ were used to infect DEFs cultured in 12-well plates individually. After 36 h of transfection or infection, the cells were lysed using RIPA buffer. The lysates were centrifuged at 4 °C, 12 000 rpm for ten minutes to obtain a clarified supernatant. The supernatant was boiled at 100 °C for ten minutes, and Peptide N-glycosidase F (PNGase F) was added following the manufacturer’s instructions (P0704S, NEB). The sample was then incubated at 37 °C for one hour for enzymatic deglycosylation. The reactants were subjected to 12% SDS-PAGE and then Western blot analysis. Recombinant virus gI was detected using a mouse polyclonal antibody against DPV gI as the primary antibody, which was prepared in this laboratory. A rabbit polyclonal antibody against DPV VP5 served as a control for viral infection, and GAPDH was used as a loading control.

### Subcellular localisation of recombinant gI

For co-localisation analysis, DEFs were plated onto glass slides in 12-well plates. The following transfections or infections were conducted: (1) DEFs were co-transfected with 500 ng of various plasmids pCAGGS-gI-Flag, pCAGGS-gI_N69A_-Flag, pCAGGS-gI_N78A_-Flag, pCAGGS-gI_N265A_-Flag, pCAGGS-gI_N69/78/265A_-Flag, and 500 ng of pDsRed2-ER. (2) DEFs were co-transfected with 500 ng of pCAGGS-gI-Flag, pCAGGS-gI_N69/78/265A_-Flag, and 500 ng of pCAGGS. (3) DEFs were co-transfected with 500 ng of pCAGGS-gI_N69/78/265A_-Flag and 500 ng of pCAGGS-gE-MYC. (4) DEFs were infected with 0.01 MOI of DPV CHv△gE-gI_N69/78/265A_ and DPV CHv-gI_N69/78/265A_. After a 36-h incubation period, an indirect immunofluorescence assay (IFA) following the previously described protocol [32]. The cells were incubated with the specified primary antibody (diluted 1:200) overnight at 4 °C. This was followed by a 2-h incubation with Alexa Fluor 488-conjugated goat anti-mouse IgG (F-2761, Invitrogen) and Alexa Fluor 586-conjugated goat anti-rabbit IgG (T-2769, Invitrogen) as the secondary antibodies (diluted 1:1000). Subsequently, the cells were incubated with 4′,6-diamidino-2-phenylindole (DAPI) (D3571, Invitrogen) at room temperature for 5 min. Finally, the glass slides were examined under a fluorescence microscope.

### Co-immunoprecipitation

1000 ng of pCAGGS-gI-Flag, pCAGGS-gI_N69A_-Flag, pCAGGS-gI_N78A_-Flag, pCAGGS-gI_N265A_-Flag and pCAGGS-gI_N69/78/265A_-Flag along with 1000 ng of pCAGGS-gE-MYC were transfected into HEK 293 T cells. Following transfection for 36 h, the cells were lysed using RIPA buffer. The lysate was centrifuged at 4 °C, 12 000 rpm for ten minutes to obtain a clarified supernatant. Following the centrifugation, 0.5 mL of the cell lysate from each sample was taken and incubated with 10 μg of the specified antibody and 1 mg of SureBeads Protein G (Thermo Fisher Scientific, Waltham, MA, USA) at room temperature for 2 h. The SureBeads Protein G were washed three times with PBST (phosphate buffer solution with Tween) and centrifuged at 13 000 × *g* for 1 min. Finally, the precipitates were resolved by 12% SDS-PAGE, and a western blot was performed using appropriate antibodies. The blots were visualised using the Clarity Western ECL system.

### In vivo experiments

At 14 days of age, ducks were inoculated with 10^6^ TCID_50_ DPV CHv_50_ (*n* = 10), DPV CHv-gI_N69/78/265A_ (*n* = 10), DPV CHv-gI_N69/78/265A_ repair (*n* = 10), and MEM (*n* = 10), respectively. The ducks in each group were continuously monitored for clinical signs, including percentage survival, rectal temperature, and body weight. Afterwards, 14-day-old ducks were re-inoculated with 10^6^ TCID_50_ DPV CHv_50_ (*n* = 10), DPV CHv-gI_N69/78/265A_ (*n* = 10), DPV CHv-gI_N69/78/265A_ repair (*n* = 10), and MEM (*n* = 10), respectively. Infected ducks were regularly autopsied, internal organs were collected, and micropathological sections were made to observe the histopathological changes of different organs. At the end of the experiment, all surviving ducks were humanely euthanised.

### Quantification of DPV gene copy number

The genome copy number of DPV in duck viscera was measured to determine the viral load. Samples of heart, liver, spleen, glandular stomach, duodenum, cecum, bursa of Fabricius, and thymus were collected from infected animals at 2, 5, 8 and 10 days after infection. Virus replication in vivo was monitored by quantifying the DPV gene copy number. DNA was extracted from all samples using a cell and tissue genomic DNA extraction kit following the manufacturer’s instructions (Magen; Catalog number: D3191-03). The DPV genome copy number was detected by qPCR [32].

### Data analysis

All experiments were conducted three times, and statistical analysis was performed using GraphPad Prism 9. Virus plaque size data was analysed using one-way analysis of variance (ANOVA). The survival curve of ducks was analysed using the logarithmic rank (Mantel-Cox) test. The viral load of recombinant virus in different tissues was analysed using a *t*-test. The *P* value of less than 0.05 was considered statistically significant.

### Ethics statement

All one-day-old ducks were purchased from a farm operated by Sichuan Agricultural University (Sichuan, China). The ducks were free of DPV and tested negative for DPV antibodies. They were housed in the animal facilities at Sichuan Agricultural University in Chengdu, China. This study was approved by the Experimental Operation Guidelines and Animal Welfare Committee of Sichuan Agricultural University (Approval license No. XF2014-18).

## Results

### Conservative analysis of gI N-glycosylation sites in *Alphaherpesvirinae*

**Figure 1:**
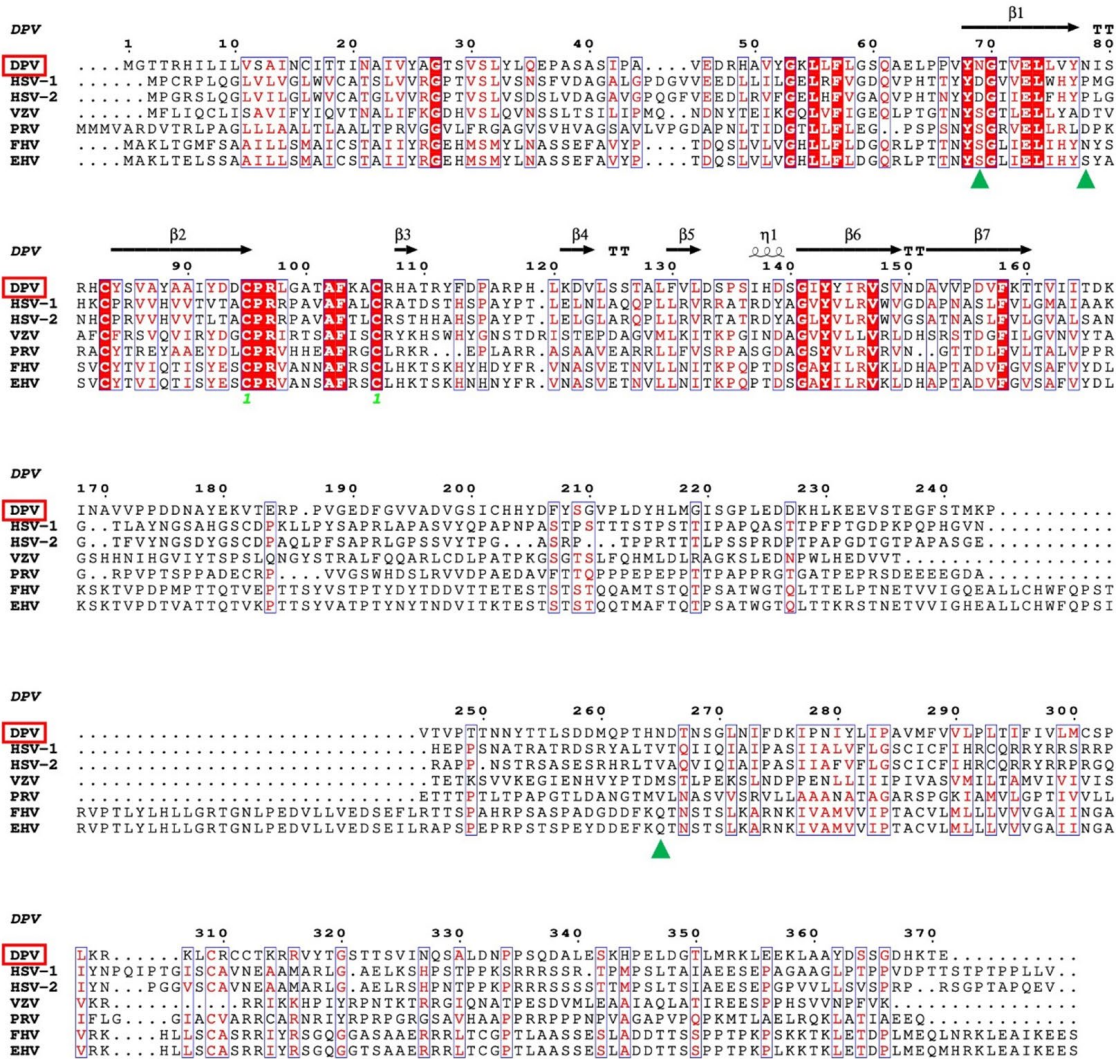
**Based on the secondary structure of DPV gI, the sequence alignment of the Alphaherpesvirus gI sequence was conducted.** Overall, conserved residues are represented with a red background, highly conserved residues are indicated by red characters, and regions with a high degree of conservation are framed in blue. The secondary structure of DPV gI is displayed above the alignment, with α-helices represented as small wavy lines, beta-strands depicted as arrows, and strict β-turns denoted by the letter “T” in a TT configuration. Conserved disulfide bonds are indicated below the sequence with green numerals. Green triangles denote potential N-glycosylation sites. The accession numbers for the *Alphaherpesvirus* gI sequences are as follows: Herpes simplex virus type 1 (HSV-1), AAW49017.1; Herpes simplex virus type 2 (HSV-2), AWP48734.1; Varicella-zoster virus (VZV), AAK01046.1; Pseudorabies virus (PRV), UQK92472.1; Feline herpesvirus (FHV), WMD92286.1; Equine herpesvirus (EHV), YP_010795113.1.

The detailed alignment of multiple sequences of gI showed differences between species in predicting N-glycosylation sites in the *Alphaherpesvirinae*. Protein N-glycosylation is a post-translational modification that usually happens on the N-X-(S/T) consensus sequence, where X is any amino acid except proline. However, not all N-X-(S/T) sequences are glycosylated, indicating that while the N-X-(S/T) motif is necessary for protein glycosylation, it is insufficient. A comparative analysis of the primary structures of gI from six *Alphaherpesvirinae* species, as shown in Figure 1, reveals that all species have three or more predicted N-glycosylation sites, but these sites are not conserved. DPV gI is a type I transmembrane glycoprotein consisting of an extracellular domain (ETD), a transmembrane domain (TMD), and a cytoplasmic domain (CTD). Its ETD is rich in asparagine residues and contains three potential N-glycosylation consensus sequences. This analysis emphasises the complexity and variability of N-glycosylation in *Alphaherpesvirinae* and stresses the need for a deeper understanding of the role of glycosylation in viral pathogenesis.

### Generation of recombinant viruses with mutations at each potential N-glycosylation site on the DPV gI through the DPV genetic operating system

**Figure 2:**
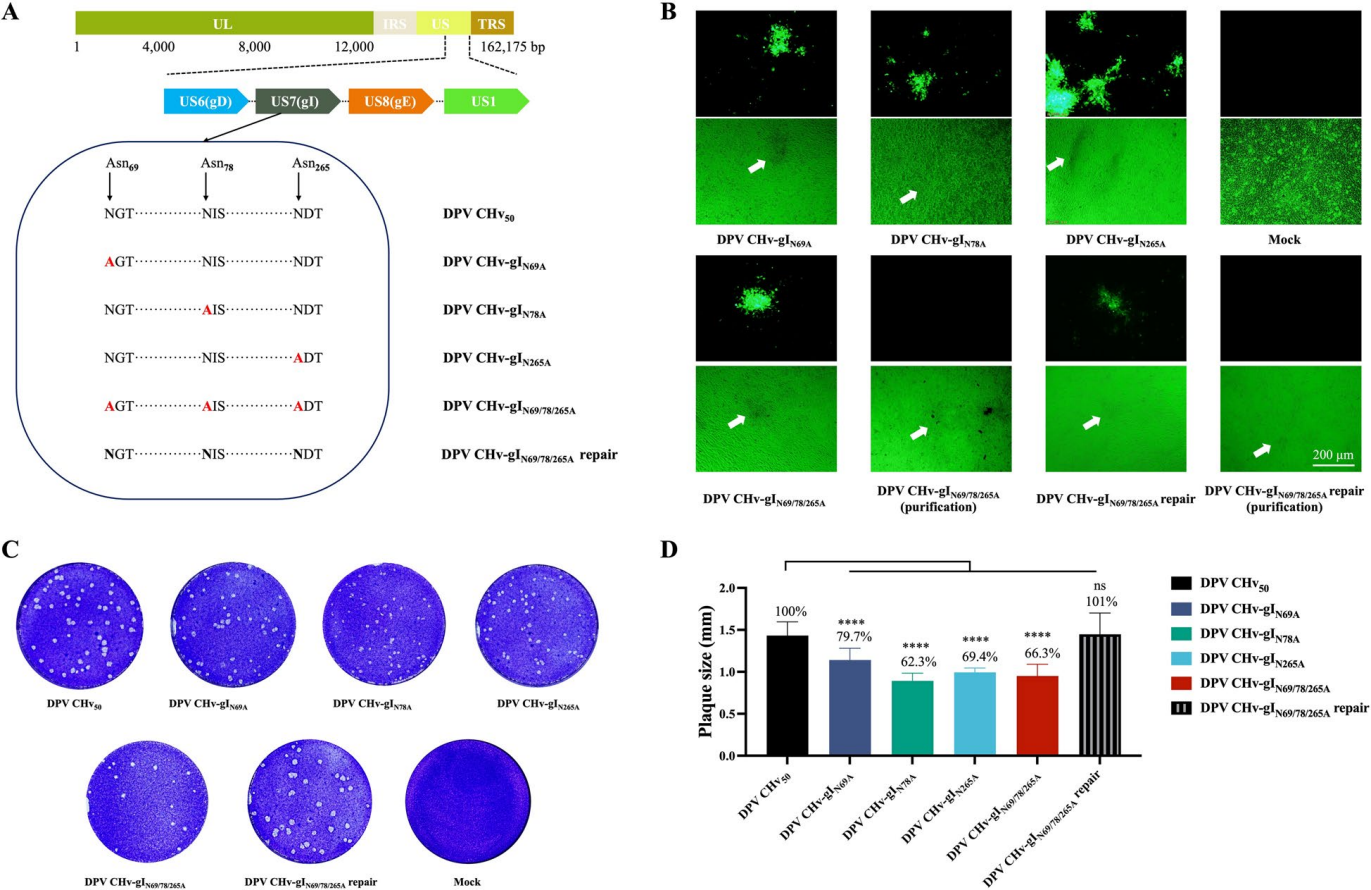
**Recombinant virus rescue and extracellular characterisation of the gI N-glycosylation site mutation in the DPV CHv strain.** **A** Schematic representation of the gI N-glycosylation site mutation in the DPV CHv strain. **B** Following the transfection of plasmids containing the gI mutational infectious clones (pDPV CHv-gI_N69A_, pDPV CHv-gI_N78A_, pDPV CHv-gI_N265A_, pDPV CHv-gI_N69/78/265A_, pDPV CHv-gI_N69/78/265A_ repair, and pDPV CHv△gE-gI_N69/78/265A_), the successful rescue of the virus was indicated by the observation of EGFP under a fluorescence microscope, while CPE was monitored using a light microscope. Cells transfected with the mock plasmid served as the control group. **C** Crystal violet staining was utilised to assess viral plaques. **D** Fifteen plaques were randomly selected per group for measurement of plaque diameter using Image J software (ns indicates not significant, *P* > 0.05; *, *P* < 0.05; **, *P* < 0.01; ***, *P* < 0.001; ****, *P* < 0.0001; *t*-test).

DPV gI has potential N-glycosylation sites at the following locations: Asn_69_, Asn_78_, and Asn_265_. We created recombinant viruses to study how gI N-glycosylation affects recombinant viruses and their impact on ducks, as shown in Figure 2A. Using the DPV genetic operating system, we employed a two-step recombination mediated by Red. This process involves the first recombination for inserting PCR amplification of the selective marker and a second recombination for removing the Kan insertion marker using the *I-Scel* cutting step. The system has been widely used for marker-free modification of large DNA molecules, such as the duck plague virus genome cloned into *Escherichia coli* bacterial artificial chromosome (BAC).

In Figure 2B, all viruses displayed green fluorescence (EGFP) following transfection. The gI gene of the recombinant virus underwent sequencing, and comparison with the DPV CHv strain genome confirmed the absence of additional mutations in the F1 generation. These findings suggest that all recombinant viruses, including DPV CHv-gI_N69A_, DPV CHv-gI_N78A_, DPV CHv-gI_N265A_, DPV CHv-gI_N69/78/265A_, DPV CHv-gI_N69/78/265A_ repair, and DPV CHv△gE-gI_N69/78/265A_, were successfully rescued.

### The effects of mutations at each potential gI N-glycosylation site on the electrophoretic mobility in both the presence and absence of PNGase F

**Figure 3:**
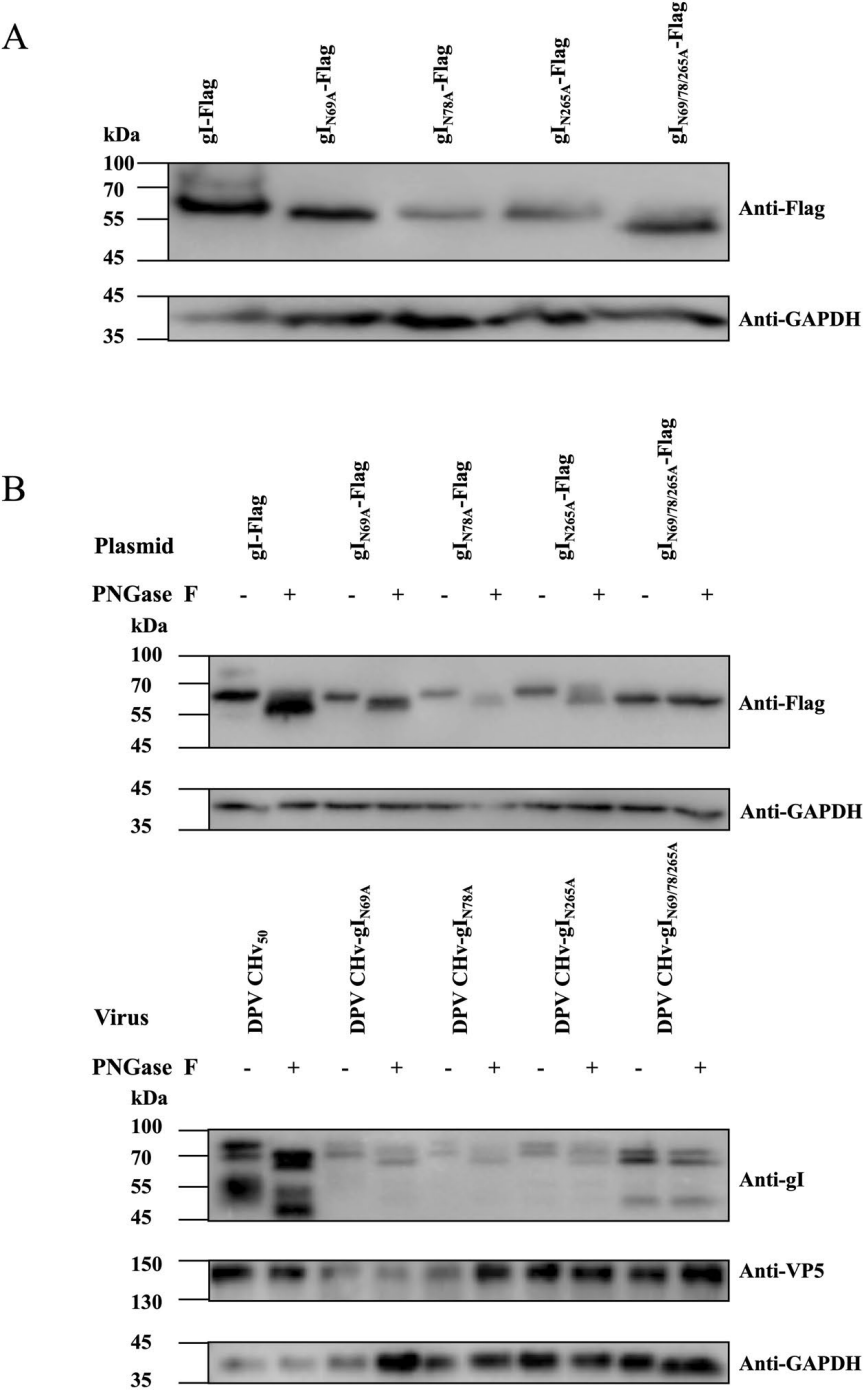
**Recombined gI MW and deglycosylation analysis.** (A) DEFs were transfected with gI expression plasmids (pCAGGS-gI-Flag, pCAGGS-gI_N69A_-Flag, pCAGGS-gI_N78A_-Flag, pCAGGS-gI_N265A_-Flag, and pCAGGS-gI_N69/78/265A_-Flag) at a dose of 1000 ng per well. Western blot analysis was performed on cell lysates 36 h post-transfection, and recombinant gI proteins were detected using a mouse monoclonal anti-Flag antibody: gI-Flag (lane 1), gI_N69A_-Flag (lane 2), gI_N78A_-Flag (lane 3), gI_N265A_-Flag (lane 4), and gI_N69/78/265A_-Flag (lane 5). (B) Collect the cell lysates from DEFs transfected with recombinant gI plasmids, and treat them with PNGase F at 37 °C for 1 h. The digested products of PNGase F are then subjected to 12% SDS-PAGE, followed by western blot. Recombinant gI is analysed using a mouse monoclonal anti-Flag antibody, with GAPDH serving as a loading control. Similarly, cell lysates from DEFs infected with recombinant viruses were processed in the same manner. Analysis of recombinant gI using a mouse polyclonal anti-gI antibody, with a rabbit polyclonal anti-VP5 antibody serving as a control for viral infection, and GAPDH as a loading control. The results presented are representative of three independent experiments.

To compare the molecular weight (MW) differences of each recombinant gI, we analysed cell lysates from the transfected cells using Western blot. Due to the addition of multiple Flag tags, the MW of the detected gI-Flag is about 70 kDa, which is higher than predicted (Figure 3A, Lane 1). However, all modified gI proteins show lower MW and the gI_N69/78/265A_-Flag migrates more quickly than individual mutants, including gI_N69A_-Flag, gI_N78A_-Flag, and gI_N265A_-Flag (Figure 3A, Lanes 2, 3, 4 and 5).

Cell lysate was collected, treated with or without PNGase F, and analysed by western blot. The results in Figure 3B show that all gI mutants migrate faster than gI in denaturing gels. After treating the infected cell lysates with PNGase F, all gI mutants exhibited a migration rate in denaturing gels that was as slow as gI. We observed the same pattern in the natural viral infection and the overexpression form through plasmid transfection. The levels of all gI mutants detected by Western blot were similar to those of wild-type gI. These results suggest that gI is glycosylated at each of the three potential N-glycosylation sites without affecting the virus accumulation in infected cells.

### Deglycosylation affects the subcellular localisation of recombinant gI

**Figure 4:**
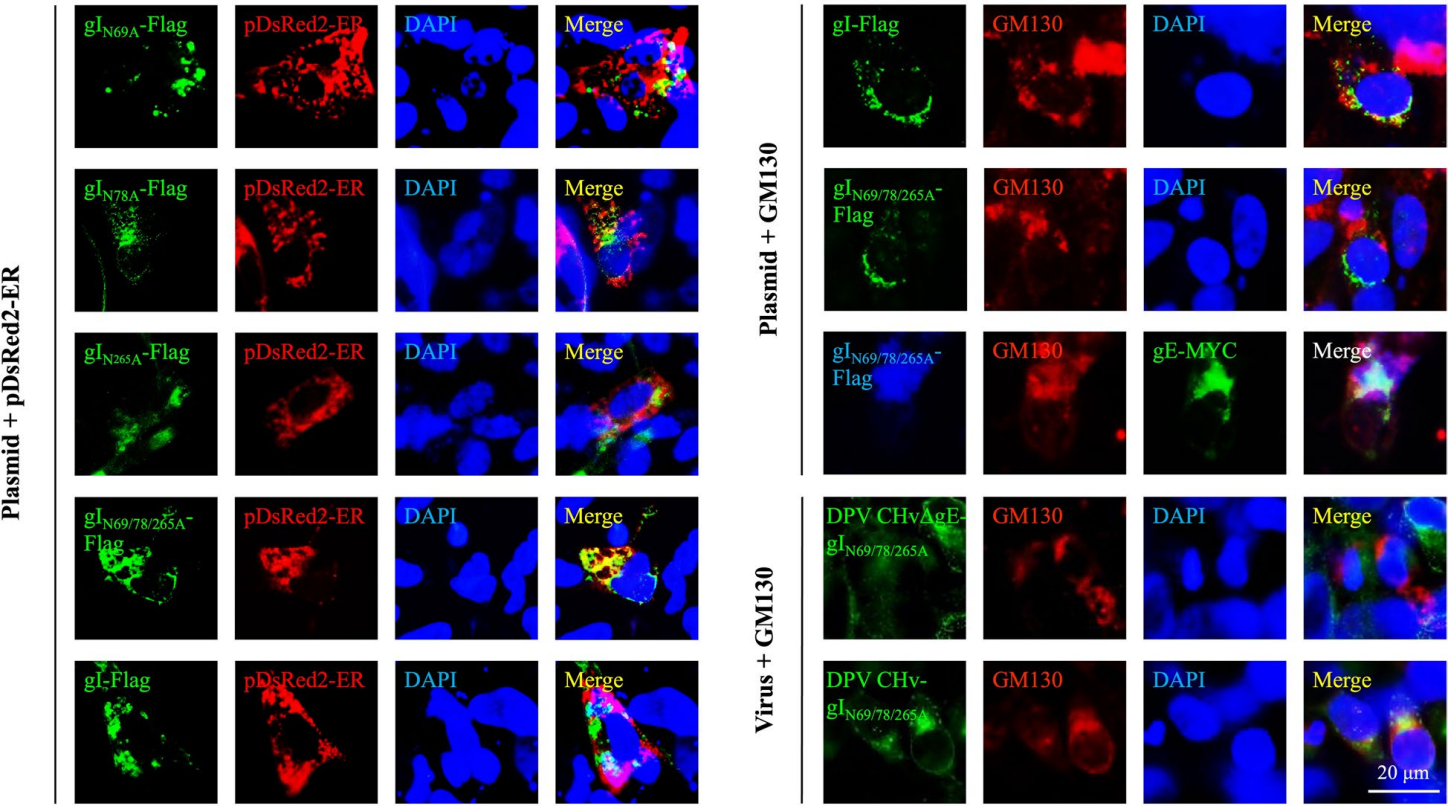
**Subcellular localisation of recombinant gI protein.** (1) The recombinant gI plasmids (pCAGGS-gI-Flag, pCAGGS-gI_N69A_-Flag, pCAGGS-gI_N78A_-Flag, pCAGGS-gI_N265A_-Flag, and pCAGGS-gI_N69/78/265A_-Flag) were co-transfected with pDsRed2-ER into DEFs. (2) The pCAGGS-gI-Flag and pCAGGS-gI_N69/78/265A_-Flag plasmids were individually transfected into DEFs, while the pCAGGS-gI_N69/78/265A_-Flag and pCAGGS-gE-MYC plasmids were co-transfected into DEFs, and their localisation was marked using GM130. (3) DPV CHvΔgE-gI_N69/78/265A_ and DPV CHv-gI_N69/78/265A_ were respectively used to infect DEFs, and their localisation was similarly marked using GM130. Transfection or infection was carried out, and IFA was performed after 36 h. Fluorescence microscopy was used for visualisation (magnification: 600 × ; scale bar: 20 μm).

During biosynthesis, membrane proteins initially receive a mannose-rich core polysaccharide precursor in the endoplasmic reticulum and then develop into heterozygous or complex oligosaccharides in the Golgi apparatus. We wanted to find out if gI mutants gather in the cellular compartments of the secretory pathway. To investigate this, we co-labelled DEFs with Flag antibody and an ER-specific fluorescent marker. The Flag antibody can mark both gI and its mutant forms. The results showed that gI single N-glycosylation site mutant did not co-locate with ER in DEFs, but gI_N69/78/265A_-Flag co-located with ER in DEFs when all of its N-glycosylation sites were mutated. Does mutation of all N-glycosylation residues reduce the surface levels of gI in DEFs? Our observations showed that the localisation of gI_N69/78/265A_-Flag and the Golgi marker protein GM130 in DEFs was severely impaired. However, we found that gI_N69/78/265A_-Flag could co-localise with GM130 only in the presence of gI’s complex partner gE (Figure 4). In conclusion, our data on the subcellular localisation confirmed that the subcellular positioning of recombinant gI was affected only after all N-glycosylation sites were mutated. The mutated gI is mistakenly retained within the internal secretory compartments, causing transport defects. However, its interacting protein, gE, can carry deglycosylated gI to the Golgi compartment.

### gI/gE complex formation is not affected by the mutation of the gI N-glycosylation site

**Figure 5:**
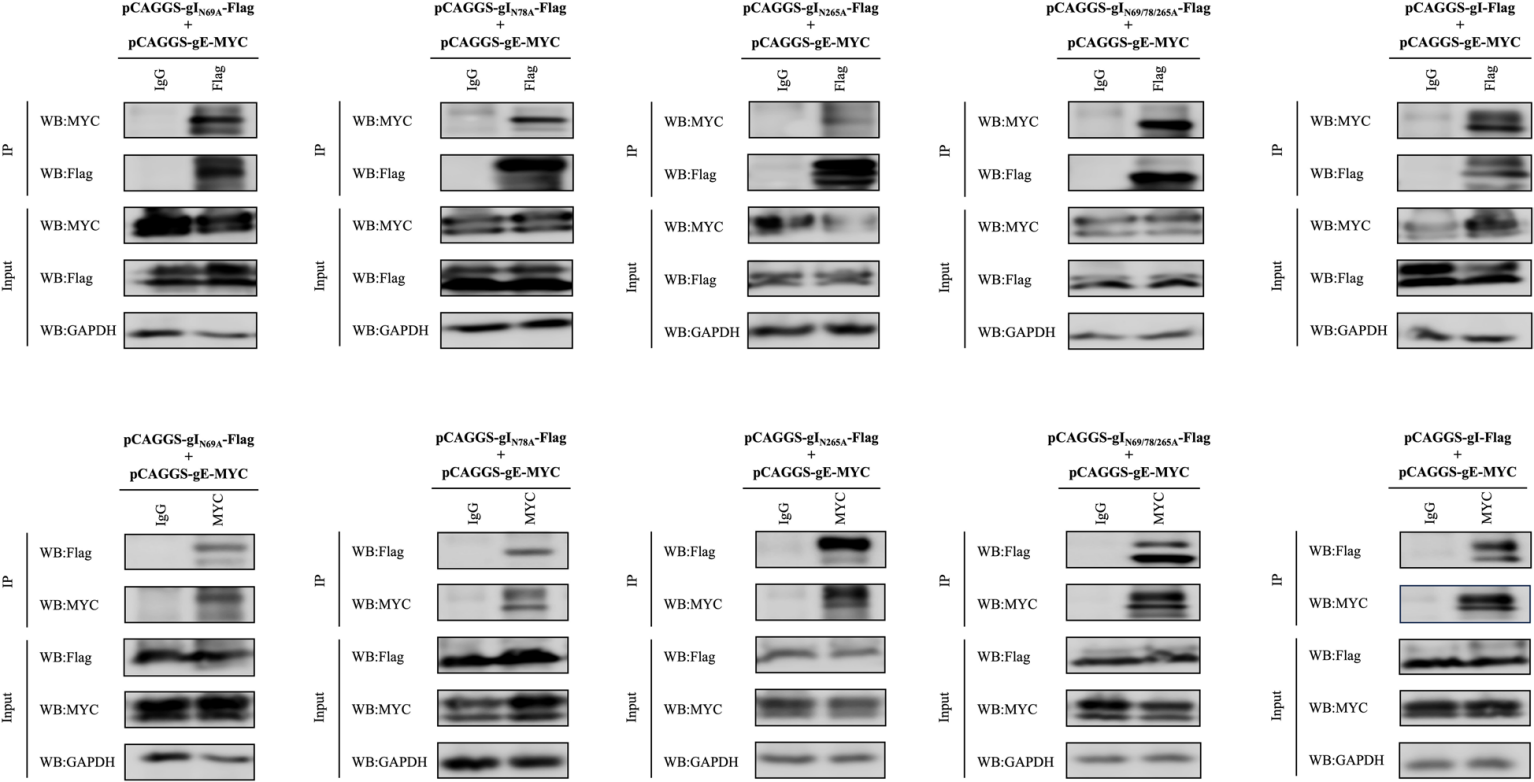
**CO-IP was utilised to examine the interaction between gI and gE after the deglycosylation of gI.** The recombinant gI plasmids (pCAGGS-gI-Flag, pCAGGS-gI_N69A_-Flag, pCAGGS-gI_N78A_-Flag, pCAGGS-gI_N265A_-Flag, and pCAGGS-gI_N69/78/265A_-Flag) with the pCAGGS-gE-MYC plasmid and cotransfect them into HEK 293 T cells for 36 h. For the immunoprecipitation (IP) process, the Anti-Flag antibody was utilised for the direct capture of interacting proteins, while the Anti-MYC antibody was employed for the reverse capture of interacting proteins. IgG antibody was used as a mock control to ensure specificity. For immunoblotting (IB), anti-Flag and anti-MYC antibodies were applied to detect the presence of the targeted proteins. The results presented are representative of three independent experiments.

N-glycosylation is crucial in various cellular protein structure and function processes, including protein–protein interactions. In herpesvirus, gI typically forms a complex with gE, which is involved in virus intercellular transmission and assembly. To investigate the impact of mutations at the gI N-glycosylation site on the subcellular localisation of recombinant gI, we observed that gI_N69/78/265A_-Flag could only co-locate with GM130 in DEFs in the presence of gE. This led us to speculate that the gI N-glycosylation site mutation would not disrupt the formation of the gI/gE complex. To verify this speculation, we conducted immunocoprecipitation experiments, which showed that mutations at the gI N-glycosylation site, whether single or total, did not affect the formation of gI/gE complex (Figure 5). IFA experiments also yielded the same result (data not shown).

### Exogenous growth kinetics of gI N-glycosylation site mutants

In our study, we investigated the size of viral plaques. We found that the diameter of plaques formed by cells infected with gI N-glycosylation site mutants was significantly smaller than that of cells infected with DPV CHv_50_. We randomly selected 15 plaques from each group, measured their diameter using ImageJ software, and then performed statistical analysis using GraphPad Prism 9 software. Our results, shown in Figures 2C, D, indicated that the plaque diameter formed by DPV CHv-gI_N69/78/265A_ was about 66.3% of that of DPV CHv_50_. We also observed that point mutants of gI N-glycosylation affected the plaque diameter of infected cells. Based on these findings, we tentatively concluded that gI N-glycosylation is involved in the late stage of DPV replication.

### gI N-glycosylation promotes the pathogenesis and lethality of duck plague

**Figure 6:**
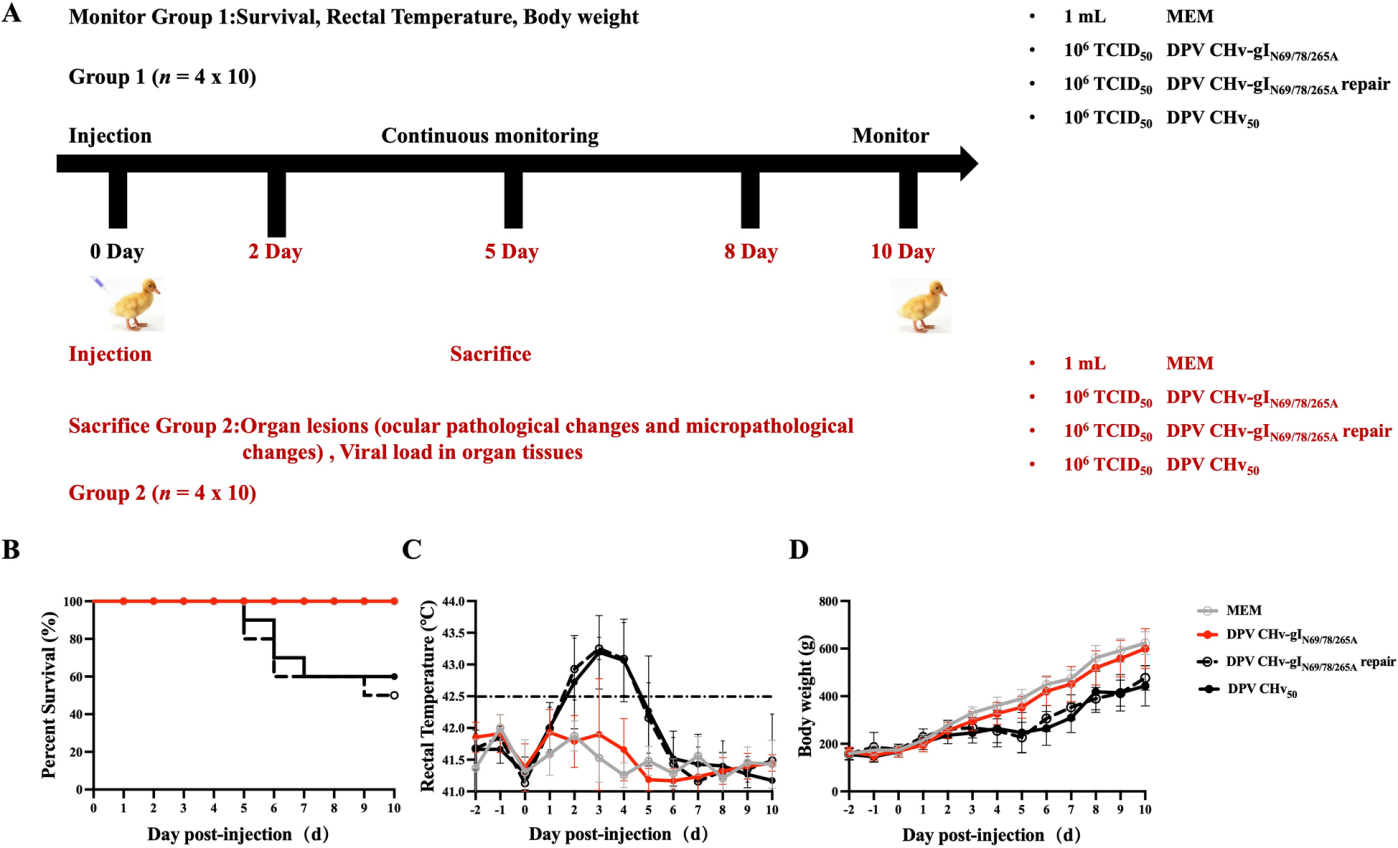
**In vivo experiments.** **A** Schematic representation of the experimental procedure investigating the impact of gI N-glycosylation on DPV. At 14 days old, ducks were intramuscularly injected with DPV CHv_50_, DPV CHv-gI_N69/78/265A_, DPV CHv-gI_N69/78/265A_ repair, and MEM (10^6^ TCID_50_). Ducks injected with MEM served as negative controls. Group 1 (*n* = 40, 10 ducks per group) was used to determine the survival rate, rectal temperature, and body weight of the ducks. Group 2 (*n* = 40, 10 ducks per group) was euthanised at 2, 5, 8, and 10 days post-inoculation to assess pathological changes in different organs and viral load. **B**–**D** Survival curves (**B**), rectal temperature (**C**), and body weight (**D**) of ducks inoculated with the specified viruses. The data were analysed using the Mantel-Cox log-rank test.

To investigate whether gI N-glycosylated mutants play a role in the pathogenesis of DPV, we infected 14-day-old ducks with the virus at a concentration of TCID_50,_ as shown in Figure 6A. We then observed the infected ducks for ten consecutive days. In the DPV CHv-gI_N69/78/265A_ group, only two ducks had rectal temperatures exceeding 42.5 °C on the third day post-challenge. The rest of the ducks maintained body temperatures, albeit fluctuating, within the normal range throughout the monitoring period, and their body weight increased steadily at an average daily gain of 3.61 g per duck. No mortality was recorded in this group. In the control group (MEM), all ducks maintained body temperatures within the normal range, consistently increasing body weight at an average daily gain of 3.86 g per duck, and no mortality occurred. In the DPV CHv_50_ group, nearly all ducks had rectal temperatures exceeding 42.5 °C post-inoculation from day one to five. Some ducks' temperatures even rose above 43.5 °C. Between the second and sixth days, there was no significant increase in body weight. However, after the sixth day, body weight continuously increased. Mortality was first observed on the fifth-day post-inoculation, with the peak of mortality occurring on the sixth day, during which two ducks died. Four ducks died during the monitoring period, resulting in a mortality rate of 40%.

To eliminate the effects of non-target mutations, we used DPV CHv-gI_N69/78/265A_ repair to infect ducks. The rectal temperature exceeded 42.5 °C from days two to five, and the body weight hardly increased during this period. However, it continued to increase after day five. Mortality was observed on the 5^th^ day post-infection, with a total of five deaths recorded during the monitoring period, resulting in a mortality rate of 50% (Figures 6B–D). The data indicated that gI N-glycosylation mutants could somewhat reduce the virus virulence.

### gI N-glycosylation mutation inhibits DPV-induced pantropic tissue damage and its replication in vivo

**Figure 7:**
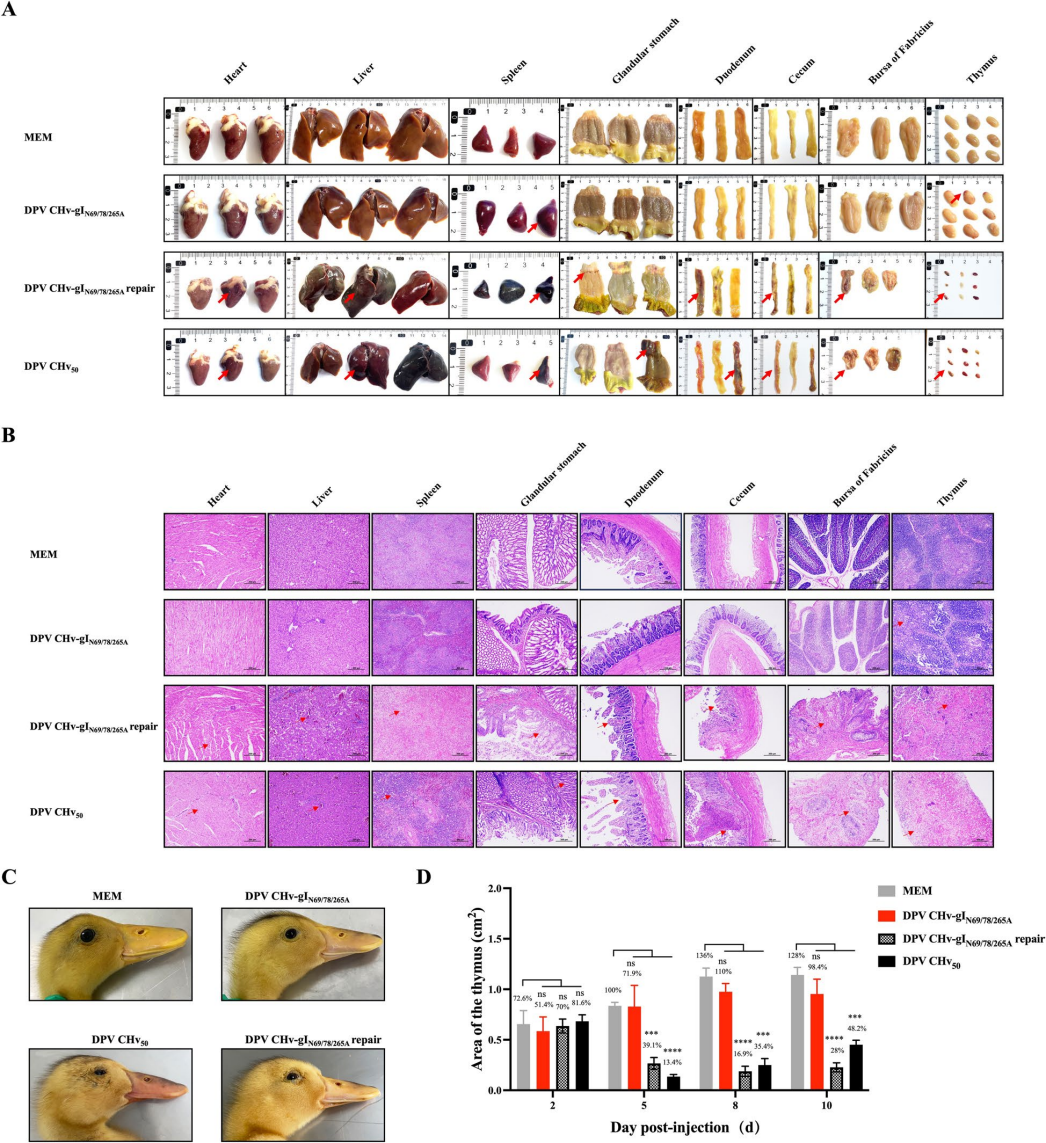
**Autopsy histopathology of designated viruses-infected ducks**. **A** At day 5 post-infection with the designated viruses (DPV CHv_50_, DPV CHv-gI_N69/78/265A_, DPV CHv-gI_N69/78/265A_ repair, and MEM), ducks were subjected to necropsy, and histopathological lesions in various tissues were documented. **B** Histological analysis of the heart, liver, spleen, glandular stomach, duodenum, cecum, bursa of Fabricius and thymus after infection with designated viruses. (haematoxylin and eosin staining, 100 × magnification). **C** Records of the characteristic features of ducks' heads during the disease phase following infection with the designated viruses. (D) Infection with DPV CHv_50_ leads to atrophy of the thymus. Each group's thymus area of ducks was scanned and measured using ImageJ. The results were calculated based on the thymus area of the control group ducks on the 5^th^ day as 100% (ns, *P* > 0.05; *, *P* < 0.05; **, *P* < 0.01; ***, *P* < 0.001; ****, *P* < 0.0001; *t*-test).

Next, to address whether gI N-glycosylation mutations inhibit DPV replication in vivo, we monitored tissue lesions and viral loads in infected animals throughout the experiment. Death occurred in the DPV CHv_50_ and DPV CHv-gI_N69/78/265A_ repair-infected ducks. Postmortem observations showed that the dead ducks showed systemic sepsis symptoms, including bleeding spots on the heart surface, severe liver congestion and necrosis, spleen enlargement, bleeding and soft texture, glandular stomach, duodenum, cecum, bursa of Fabricius and thymus haemorrhage and atrophy. However, there were no deaths in the DPV CHv-gI_N69/78/265A_ group. Anatomical observation of surviving ducks showed spleen haemorrhage and enlargement, slight haemorrhage of the thymus, and no obvious lesions in other organs (Figure 7A). The histopathological section results showed that in the DPV CHv-gI_N69/78/265A_ group, infected ducks' tissues such as the heart, liver, spleen, glandular stomach, duodenum, cecum, and bursa of Fabricius did not exhibit significant lesions compared to the MEM group, with minor reticular necrotic areas observed in the thymus tissue. In contrast, ducks infected with DPV CHv_50_ and the DPV CHv-gI_N69/78/265A_ repair group exhibited myocardial fibre rupture in the heart; congestion in the hepatic tissue vessels and sinusoids, with severe fatty degeneration of the liver tissue; areas of necrosis in the spleen devoid of cellular components, presenting a reticular pattern; necrosis of the deep tubular glands in the glandular stomach; mucosal epithelial shedding and villous breakage in the duodenum and cecum; indistinct corticomedullary boundaries in the bursa of Fabricius; and thymic lymphocyte necrosis and fragmentation, filled with a large amount of pale red interstitial tissue and red blood cells (Figure 7B). During the monitoring process, it was observed that the head and neck of ducks in the DPV CHv_50_ and DPV CHv-gI_N69/78/265A_ repair infected group were enlarged, and the eyelids were soaked, showing typical symptoms of “Big Head Plague” (Figure 7C). The ducks in the DPV CHv-gI_N69/78/265A_-infected group showed no obvious changes in the appearance of the head as in the MEM group. Interestingly, in the later stages of viral infection, the thymus of ducks in the DPV CHv_50_ group shrank to only 13.4% to 35.4% of those in the MEM group (Figure 7D). These results suggest that gI N-glycosylation is involved in the pathogenesis of DPV, which partially prevents DPV-induced thymus atrophy.

The virus replication in different tissues and organs of infected ducks was measured using qPCR at various time points. On the fifth day post-infection, the range of viral copy numbers in multiple organs of the DPV CHv-gI_N69/78/265A_ group ducks was from 10^5.62^ to 10^7.39^ copies/0.1 g, with the highest viral content found in the thymus. The viral content in various organs of ducks in the DPV CHv_50_ and DPV CHv-gI_N69/78/265A_ repair groups was generally higher than that in the DPV CHv-gI_N69/78/265A_ group. The range of viral copy numbers in the organs of ducks in the DPV CHv_50_ group was from 10^7.94^ to 10^10.17^ copies/0.1 g, while the range of viral copy numbers in the organs of ducks in the DPV CHv-gI_N69/78/265A_ repair group was from 10^7.78^ to 10^10.11^ copies/0.1 g. At this point, the average viral copy number in the DPV CHv-gI_N69/78/265A_ group was 100.1 times lower than that in the DPV CHv_50_ group and 92.5 times lower than that in the DPV CHv-gI_N69/78/265A_ repair group.

**Figure 8:**
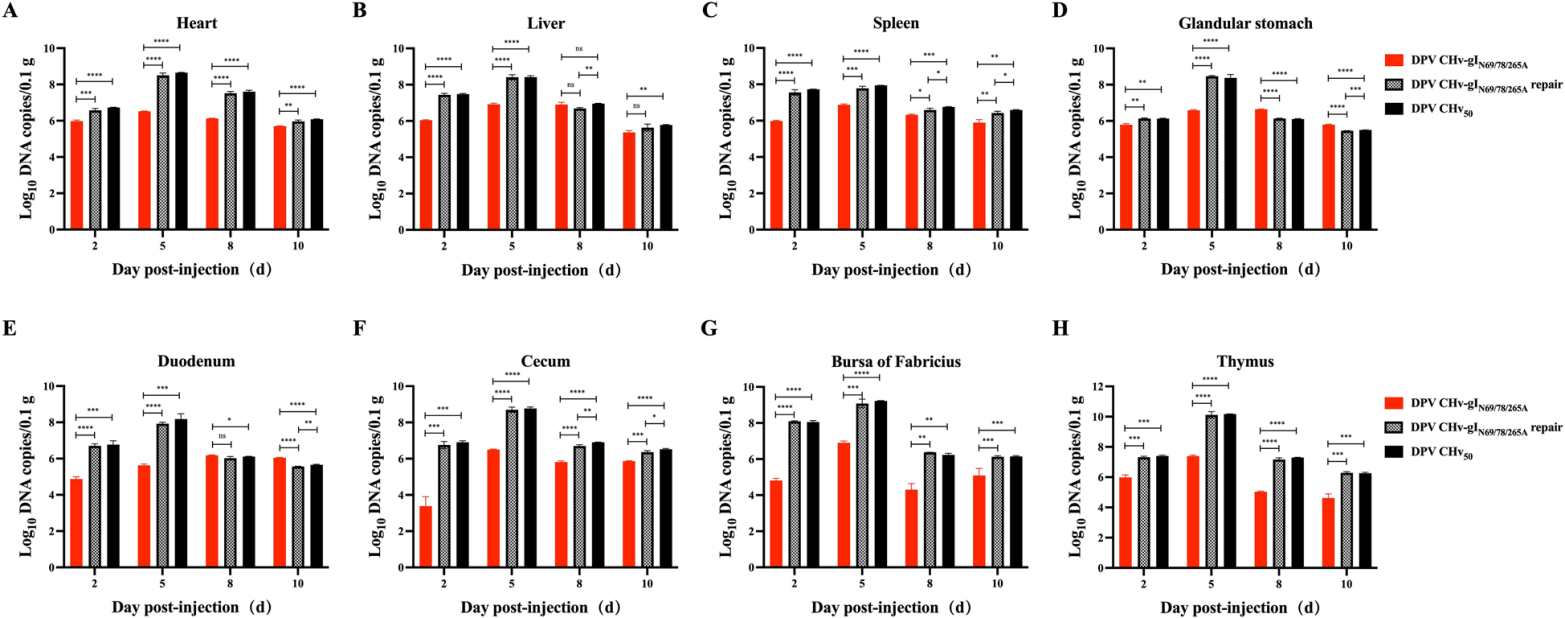
**Replication characteristics of gI N-glycosylation mutant recombinant virus in ducks.** **A**–**H** Quantitative determination of the designated virus DPV genomic copy number in the heart (**A**), liver (**B**), spleen (**C**), glandular stomach (**D**), duodenum (**E**), caecum (**F**), bursa of Fabricius (**G**), and thymus (**H**) of experimentally infected ducks (ns, *P* > 0.05; *, *P* < 0.05; **, *P* < 0.01; ***, *P* < 0.001; ****, *P* < 0.0001; *t*-test)5ʹ.

On the tenth day post-infection, there was a significant decrease in viral copy numbers in the organs of ducks across all groups. The range of viral copy numbers in the organs of the DPV CHv-gI_N69/78/265A_ group was from 10^4.3^ to 10^6.04^ copies/0.1 g. In the DPV CHv_50_ group, the organ’s viral copy numbers in the organs ranged from 10^5.5^ to 10^6.58^ copies/0.1 g. In the DPV CHv-gI_N69/78/265A_ repair group, the organ’s viral copy numbers ranged from 10^5.46^ to 10^6.42^ copies/0.1 g. At this time, the average viral copy number in the DPV CHv-gI_N69/78/265A_ group was 3.2 times lower than that in the DPV CHv_50_ group and 2.5 times lower than that in the DPV CHv-gI_N69/78/265A_ repair group (Figures 8A–H).

Since antibodies have been produced in the ducks, each group of surviving ducks at this time has some resistance to death. In general, the replication level of DPV in ducks after gI N-glycosylation mutation was lower than that in the DPV CHv_50_ and DPV CHv-gI_N69/78/265A_ repair group. The decrease in viral replication ability was one of the reasons for the absence of disease and death in ducks. Therefore, N-glycosylation of gI plays a role in the function of gI as a virulence factor.

## Discussion

Glycoprotein I (gI) is encoded by the US7 gene and plays various roles in viral infection. It has been reported that gI forms a heterodimeric complex with glycoprotein E (gE) on the cell membrane, modulating the translocation of progeny viruses to intercellular junctions and directly participating in cell-to-cell spread [33, 34]. gI utilises endoplasmic reticulum (ER)-associated degradation components Derlin-1 and Sec61 to facilitate the ubiquitination of Toll-like receptors 3 (TLR3) and 4 (TLR4), thereby assisting the virus in evading host immune surveillance [35]. The interaction between gI and gamma-secretase modulates the excessive phosphorylation of tau protein and beta-amyloid (Aβ), thereby elevating the risk of neurodegenerative disorders [36]. Additionally, gI acts as a virulence gene, reducing the virulence of the virus to the host when deleted in many herpes viruses [37–39]. However, the function of gI as a glycoprotein in viral infection remains unclear.

Glycoproteins can capture N-linked and O-linked glycans. Previous studies have shown that by using an O-Glycosidase & Neuraminidase Bundle, β1-4 Galactosidase-S, and β-N-Acetylglucosaminidase S to cleave O-glycosidic sugar chains, and PNGaseF to cleave N-glycosidic sugar chains, the occurrence of O-glycosylation and N-glycosylation of DPV gI can be determined based on the changes in protein size. At the plasmid level, the MW of gI only changes upon cleavage with PNGaseF. Thus, we focused on exploring the role of N-glycosylation of gI in virulence during viral infection. We generated recombinant DPV viruses with N-glycosylated mutations using a reverse genetic system and demonstrated that N-glycosylation of DPV gI affects viral replication and pathogenesis in vivo*.*

When comparing the gI gene sequences of Alphaherpesviruses, we found that the expected N-glycosylation sites in the DPV gI were not conserved. However, in our secondary structure model, N_69_ and N_78_ are positioned at the ends of the protein surface β-strand regions. At the same time, N_265_ is buried near the lipid membrane, all within conserved structural folds. The N_620_ residue of VZV gB corresponds to the N_636_ residue of PRV. Both are located within the conserved DIV β-strand and are part of the epitope recognised by neutralising antibody (93 k) against VZV gB [40, 41]. KSHV gH ELEFN_50-54_ is also a potential glycosylation site within the presumed β-hairpin of the KSHV gH/gL interaction region. When ELEFN_50-54_ is mutated to ELAAN, glycosylation is reduced, leading to decreased binding with EphA2 and a subsequent reduction in fusion [42]. N-glycosylation sites within SAV glycoprotein are not highly conserved. E_135_N is located on the surface area within the predicted central β-strand, while E_2319_N is embedded near the lipid membrane [43]. Many flaviviruses possess highly conserved N-glycosylation sites at positions Asn_130_ and Asn_207,_ which play a role in mouse viral replication and neurovirulence. These sites are also situated at the terminal regions of the β-strand [44]. This strongly supports DPV gI N_69_, N_78_, and N_265_ as functional N-glycosylation sites.

N-glycosylation is a common post-translational modification that is essential for the localization and secretion of proteins, enabling them to perform their functions at the appropriate location. This is particularly crucial for virulence genes [45], as abnormal glycosylation can result in the loss of pathogenicity in infectious agents [46]. In general, removing the sugar molecules or altering the glycosylation sites due to the use of glucosidase inhibitors may cause the viral glycoproteins to fold incorrectly and be retained in the ER [47, 48]. Therefore, it is important to investigate how mutations at the glycosylation sites could impact the subcellular localisation of gI. The findings showed that mutating a single N-glycosylation site in gI did not affect its subcellular localisation. However, when the three N-glycosylation sites of gI are mutated at the same time, the gI is trapped in the ER. When present with gE, gE can interact with the deglycosylated gI and carry it to the Golgi apparatus. The analysis of viral glycoprotein glycosylation typically focuses on studying recombinant proteins. These proteins are usually produced in in cell lines with different glycosylation capabilities compared to the host cells. As a result, the glycosylation patterns of the resulting recombinant glycoproteins may differ from those of the native viral proteins [49]. We then looked into localisation of all N-glycosylation mutations of gI at the viral level, and the results are shown in Figure 4. The localisation patterns of the native viral proteins matched those of the recombinant proteins expressed from heterologous plasmids. We believe that structural changes caused by deglycosylation of gI might affect other processes within the viral life cycle, such as the assembly of viral particles, which could be detrimental for viral infection and proliferation (Figures 2C and 2D). However, the conformational changes do not hide the binding site for gE, and therefore, do not affect its ability to bind with gE (Figure 5).

It is undisputed that using animal samples to analyse the mechanisms of infection of animal pathogenic viruses is crucial for understanding their pathogenic mechanisms in animals. The gap between observations of viral infection in vitro and in vivo is significant. Therefore, evaluating in vivo samples should provide more valuable information on the mechanisms of infection than evaluating in vitro samples.

In this study, we also measured viral loads of infected ducks in different tissues: heart, liver, spleen, glandular stomach, duodenum, cecum, bursa of Fabricius, and thymus. Unlike replication on DEFs in vitro DPV CHv-gI_N69/78/265A_ (data not shown), the virus proliferation in ducks was significantly reduced. When detecting the lesions of various parenchymal organs related to the characteristics of duck plague, one of the characteristics of duck plague is the degeneration of systemic parenchymal organs [50].

Compared with DPV CHv_50_, DPV CHv-gI_N69/78/265A_ resulted in significantly lower virus copy numbers in different tissues. The most significant difference was on day five after injection. At that time, the DPV CHv_50_ infected group was near the death peak, and the ducks’ antibodies could not resist the virus replication in the body. Meanwhile, as shown in Figure 7A, the bleeding of the bursa of Fabricius and atrophic lesions of the thymus was most obvious in the immune organs of infected ducks in the DPV CHv50 group, while the lesions were not obvious in the immune organs of the DPV CHv-gI_N69/78/265A_ group.

On the 10^th^ day after injection, compared with DPV CHv_50_, DPV CHv-gI_N69/78/265A_ showed little or no difference in virus copy number in different tissues, and no death occurred at this time. The low degree of immune organ damage and the inhibition of viral replication are two reasons why the virulence was weakened, and the ducks did not die.

Live attenuated vaccines are highly effective in preventing viral infections. Recently, reverse genetics has been used to modify the viral genome, making it easier to develop these attenuated vaccines quickly. This technique relies on in-depth research on the virus key virulence genes [51]. The genomic sequence of DPV contains many non-essential regions for replication, which can be used as insertion sites for foreign genes. These regions can also act as carriers for multi-recombinant live vaccines [12, 52, 53]. For example, by inserting the P1 and 3C genes of Duck Hepatitis A virus (DHAV) between the US7 and US8 genes of Duck Enteritis Virus (DEV), a recombinant virus (rDEV-US7/US8-P13C) is produced. This recombinant virus triggers the production of neutralising antibodies against both DHAV-3 and DEV in ducks when inoculated. It protects against lethal DEV attacks and completely prevents DHAV-3 infection [54]. The duck Tembusu virus E gene was optimised based on the cloning of the infectious bacterial artificial chromosome (BAC) of the duck enterovirus vaccine pDEV-EF1. The resulting recombinant virus, rDEV-E451-dk, was well expressed in chicken embryo fibroblasts (CEFs) [55]. After inserting multiple Pseudomonas outer membrane proteins H (OmpH) between the DEV UL55-LORF11 and UL44-44.5 genes, rDEV-OmpH-UL55 and rDEV-mpH-UL44 were produced. Specific antibody levels against multiple Pseudomonas and DEV were generated after immunisation. This resulted in protection for ducks from the invasion of these two diseases [56]. The results above suggest that viral live vectors can be used not only to express viral immunogens but also to express bacterial immunogens, which can help prevent viral and bacterial infections. The complete genome of the CHv used in our study as a representative of the DPV potent strain, has been published, and its genetic background is clear [8]. Although the virulence of DPV CHv-gI_N69/78/265A_ in ducks has been reduced, it still maintains some virulence. Figures 6B and 7A show that infected ducks display symptoms such as elevated body temperature and thymus bleeding. After optimising the attenuated strain by mutating other virulence genes, it can be used as a carrier for live vaccines to create multivalent vaccines for controlling DPV and other poultry infections.

The N-glycosylation modification of the gI protein in DPV is crucial for its virulence. In this study, we identified mutant viruses that lack the N-glycosylation site on the gI protein, leading to reduced virulence in ducks. This discovery highlights the importance of N-glycosylation modification of structural proteins in determining the virulence of herpesviruses. The attenuated virus could potentially be further developed as the basis for an avian virus attenuated chimeric live vaccine. This provides a theoretical foundation for investigating the pathogenic mechanism of duck plague virus and developing potential attenuated live vaccines for DPV.

## Data Availability

The datasets used and/or analysed during the current study are available from the corresponding author upon reasonable request.
